# Towards an Optimal Design of Target for Tsetse Control: Comparisons of Novel Targets for the Control of Palpalis Group Tsetse in West Africa

**DOI:** 10.1371/journal.pntd.0001332

**Published:** 2011-09-20

**Authors:** Jean Baptiste Rayaisse, Johan Esterhuizen, Inaki Tirados, Dramane Kaba, Ernest Salou, Abdoulaye Diarrassouba, Glyn A. Vale, Michael J. Lehane, Stephen J. Torr, Philippe Solano

**Affiliations:** 1 Centre International de Recherche – Développement sur l'Elevage en zone Subhumide (CIRDES), Bobo-Dioulasso, Burkina Faso; 2 Liverpool School of Tropical Medicine, Liverpool, United Kingdom; 3 Natural Resource Institute, University of Greenwich, Chatham, Kent, United Kingdom; 4 Institut Pierre Richet, Abidjan, Côte d'Ivoire; 5 South African Centre for Epidemiological Modelling and Analysis (SACEMA), University of Stellenbosch, Stellenbosch, South Africa; 6 Institut de Recherche pour le Développement, UMR 177 IRD-CIRAD, CIRDES, Bobo-Dioulasso, Burkina Faso; International Centre of Insect Physiology and Ecology, Kenya

## Abstract

**Background:**

Tsetse flies of the Palpalis group are the main vectors of sleeping sickness in Africa. Insecticide impregnated targets are one of the most effective tools for control. However, the cost of these devices still represents a constraint to their wider use. The objective was therefore to improve the cost effectiveness of currently used devices.

**Methodology/Principal Findings:**

Experiments were performed on three tsetse species, namely *Glossina palpalis gambiensis* and *G. tachinoides* in Burkina Faso and *G. p. palpalis* in Côte d'Ivoire. The 1×1 m^2^ black blue black target commonly used in W. Africa was used as the standard, and effects of changes in target size, shape, and the use of netting instead of black cloth were measured. Regarding overall target shape, we observed that horizontal targets (i.e. wider than they were high) killed 1.6-5x more *G. p. gambiensis* and *G. tachinoides* than vertical ones (i.e. higher than they were wide) (P<0.001). For the three tsetse species including *G. p. palpalis*, catches were highly correlated with the size of the target. However, beyond the size of 0.75 m, there was no increase in catches. Replacing the black cloth of the target by netting was the most cost efficient for all three species.

**Conclusion/Significance:**

Reducing the size of the current 1*1 m black-blue-black target to horizontal designs of around 50 cm and replacing black cloth by netting will improve cost effectiveness six-fold for both *G. p. gambiensis* and *G. tachinoides*. Studying the visual responses of tsetse to different designs of target has allowed us to design more cost-effective devices for the effective control of sleeping sickness and animal trypanosomiasis in Africa.

## Author Summary

Tsetse flies transmit trypanosomes causing sleeping sickness and nagana. Controlling tsetse prevents transmission of these diseases. Insecticide impregnated targets are highly effective but are too costly. This study aims to improve the cost effectiveness of targets. Experiments were performed on three tsetse species in Burkina Faso or Côte d'Ivoire. Effects of target size, shape, and the use of netting instead of black cloth were measured. We observed that targets wider than they are high (horizontal target) killed 1.6-5x more *G. p. gambiensis* and *G. tachinoides* than vertical ones. Catches were highly correlated with the size of the target up to a target size of 0.75 m, beyond which there was no further increase in catches. Replacing the black cloth of the target by netting did not change catches, but was far cheaper. Hence reducing the size of the current 1 m×1 m black-blue-black target to an horizontal 0.75×0.5 m net blue net target will improve cost effectiveness six-fold for both *G. p. gambiensis* and *G. tachinoides*. Studying the visual responses of tsetse to different designs of target has allowed us to design more cost-effective devices for the effective control of sleeping sickness and animal trypanosomiasis in Africa.

## Introduction

Tsetse flies (Diptera: Glossinidae) infest about10 million km^2^ of sub-Saharan Africa where they transmit trypanosomes which cause Human African Trypanosomiasis (HAT; also known as sleeping sickness) and African Animal Trypanosomiasis (AAT; also known as Nagana). This complex of diseases has an important impact on health and economic development in sub-Saharan Africa [1], [2]. Tsetse are commonly divided into three, ecologically distinct groups: savannah tsetse ( = Morsitans group) which are largely responsible for transmitting the trypanosomes that cause nagana; riverine tsetse ( = Palpalis group) which play a major role the transmission of *Trypanosoma brucei spp.*, the causative agents of sleeping sickness; and forest tsetse ( = Fusca group) which, generally speaking, do not play an important epidemiological role.

Tsetse traps or their simplified two-dimensional derivative targets, when impregnated with insecticides, have constituted a central component of tsetse control campaigns in many countries in Africa [3]–[6], albeit such baits have been more used against AAT than HAT, except for a few notable exceptions [7], [8]. The reasons it has not been used more widely against HAT are several, but one of the most important is the financial and logistical cost of using baits [9]. Hence, if the method is to be more widely used, especially by communities directly afflicted by HAT, then these costs must be reduced [10].

The type of target used to control tsetse varies according to the geographical location of the operation and the target species of tsetse. However, in general targets are coloured blue and/or black [11], [12]. The use of blue in combination with contrasting colours such as white or black significantly improves landing behaviour of tsetse on targets [11], [13], [14], [15].

The shape of the target is also important for both the overall shape (horizontal versus vertical) and the patterns (e.g. banding) on the target. For example, vertical banding seems to be more effective than horizontal for some Palpalis group tsetse (e.g. *G. p. palpalis*, see [16]). For Morsitans group tsetse, horizontal oblongs elicit a stronger landing response than vertical ones, whereas vertical and horizontal oblongs seem equally attractive [17], [18].

Interactions between size and efficacy are also variable. While it is generally acknowledged that for the Morsitans group tsetse of East and South Africa, “the bigger the target the better” [19], [20], this is not the case for Palpalis group tsetse [11], [21], [22]. For example, recent studies in Kenya showed that a 90% reduction in target size only reduced the catch of *G. f. fuscipes* by 50% [22]. In this latter study, as well as other recent works on tsetse in West Africa (I. Tirados *et al*., In Press; J. Esterhuizen *et al*., In Press), size and shape comparisons always involved black targets. However in West Africa, the most used target to control tsetse has been the 1 m^2^ black-blue-black target developed by Laveissière *et al.* [11]. Consequently in this study, we investigated if this target could be improved in terms of size, shape and overall design, focusing on three major vectors of human and animal trypanosomiases in West Africa: *G. palpalis gambiensis*, *G. p. palpalis*, and *G. tachinoides*. To achieve this, taking into account previous observations on tsetse behaviour in particular their circling behaviour around targets [14], [19] and the poor ratio reported of the numbers landing compared to the numbers attracted [23], we designed several experiments to examine how (i) adding netting panels and/or varying (ii) the size and/or (iii) overall shape could improve the number of tsetse that might be attracted to the vicinity of a target, and then the proportion that subsequently contact it, either by landing on the target or colliding with it. Actually we show that smaller designs made of overall horizontal oblongs incorporating netting offer promising, more cost effective, alternatives to control tsetse of the Palpalis group and that these improved designs may lead to more sustainable control efforts because of their better cost efficiency than in the past.

## Methods

### Study areas

*G. p. gambiensis* and *G*. *tachinoides* were studied in southern Burkina Faso between November 2008 and April 2009, near the village of Folonzo (9.9°N, 4.6°W) along the Comoe river (January – April) where *G. tachinoides* is predominant. We also worked in western Burkina Faso on the Mouhoun river near the village of Solenzo (12.20°N, 4.4°W) in November, where only *G. p. gambiensis* is found. The detailed description of these study areas can be found in [24], [25]. Work was also undertaken on *G. p. gambiensis* in Orodara (11°18N, 5°27W) (South West of Burkina Faso) on the Pindia river. In Côte d'Ivoire the study was undertaken near Azaguié (05.67°N, 04.11°W) where *G. p. palpalis* is abundant.

### Measuring attractiveness and efficiency

**Figure 1:**
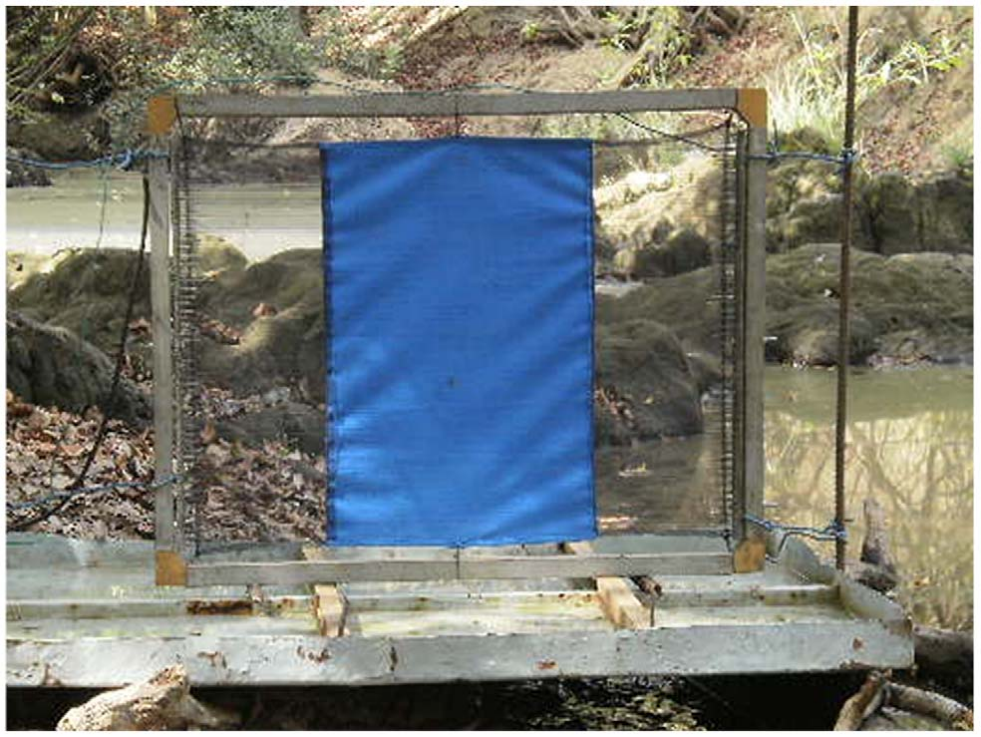
Example of a target design used for comparison. A 0.75 m wide×0.50 m high net-blue-net target (37.5 cm×50 cm blue surrounded by 2 pieces of net of 18.75 cm×50 cm each) inside an electric grid mounted on a fly collection tray.

The numbers of tsetse attracted to the vicinity of a target was assessed by covering the targets with a grid of fine electrocuting wires which killed or stunned tsetse as they landed [23]. A proportion of tsetse approaching targets do not land and to provide a relative estimate of these circling tsetse, an electrocuting net (E-net) was placed adjacent to the target. The E-net is effectively invisible to tsetse and hence circling flies collide with it. The electric targets and E- net were mounted on a tray. Tsetse contacting the electrocuting grid fall vertically into the water tray below. For instance, in the target represented in figure 1, the tray is divided in three parts to allow the separation of flies killed by contacting the blue or the two flanking nets. The combined catch from the target+E-net provided a relative measure of the number of tsetse attracted to a target.

**Figure 2:**
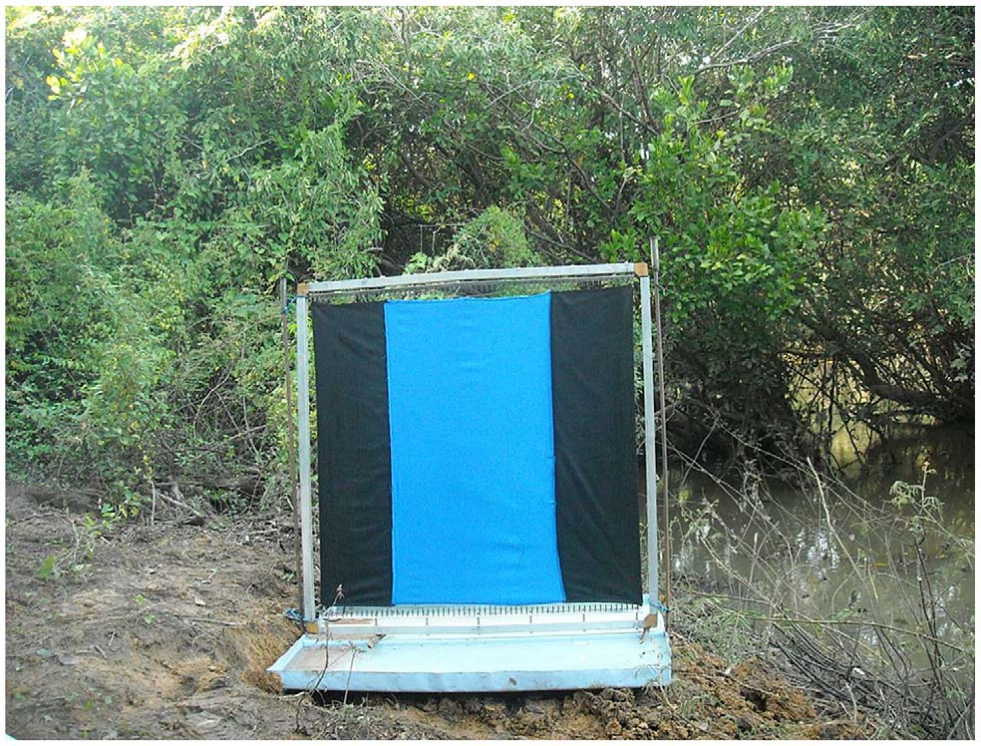
The standard 1 m×1 m Black Blue Black e-target (treatment A) set up on a tray.

Many different designs of target were studied in different experiments. To facilitate inter-experiment comparisons, a “standard” target was included in all experiments. The standard design, which is used as a control, is derived from the Laveissière *et al*. (11] black-blue-black target, and consisted of a 1 m^2^ target (1 m wide×1 m high) with three, vertical stripes of black, blue and black in widths of relative proportion 1∶2∶1, respectively (Fig. 2). Henceforth, this design will be called “the standard” in the rest of the manuscript. This design has been widely used in tsetse control operations across West Africa (see [7] for instance). The sequence of these stripes of black, blue, and black that will be found in other treatments tested here will be called “BkBlBk” in the rest of the manuscript. When the black cloth sections of a target were replaced by black netting, the letter ‘N’ was used (e.g. NBlN). Flies contact a target either by landing on the cloth or when they collide with netting panels which are invisible to them; we wanted to explore which was the best strategy to pursue in target design.

### Experimental design and analyses

All experiments were carried out for 4 h between 08∶00 hours and 12∶00 hours local time when Palpalis group species are most active [26]. The different targets were compared with the standard target in a series of replicated Latin squares of days×sites×treatments where sites were always >100 m apart.

The daily catches (*n*) were normalized and variances homogenized using a log10(*n*+1) transformation and subjected to analysis of variance using GenStat 11 edition (version 11.1.0.1504). When the ANOVA showed a significant difference after this first analysis, a Bonferroni pairwise comparison test was undertaken to detect significant differences between the different treatments. To provide a common index of the effect of shape, size or colour on catches, the detransformed mean catch of tsetse from different targets was expressed as the proportion of that from the standard target and this value was termed the catch index. For example, a target that doubles the catch from the standard target would have a catch index of 2 while one that halved the catch would have a catch index of 0.5.

Differences in the proportion of tsetse caught on different sections (e.g., blue, black or netting) of a target were analysed by logistic regression. The total catch from all sections of the target was specified as the binomial denominator and the catches from a particular section (e.g., blue section) was specified as the *y*-variable. The significance of changes in deviance was assessed by either chi^2^ or, if the data were overdispersed, an *F*-test following re-scaling [27]. The standard error is asymmetric about the mean and thus mean percentages are accompanied by the larger standard error.

Unless stated otherwise the term ‘significant’ denotes that the means differ at P<0.05.

Details of the individual experiments are given below.

### Effect of shape

Seven targets were compared, including the standard (treatment A), to determine the effect of the overall shape of the target (i.e. horizontal (wider than high) versus vertical (higher than wide)) on fly captures. The 6 targets were compared by pairs as following, with the first number indicating the width and the second one, the height (in meters): 0.5×0.75 net-blue-net (NBlN) vertical (Treatment B) *versus* 0.75×0.5 NBlN horizontal (C); 0.25×0.5 black-blue-net (BkBlN) vertical (D) *versus* 0.5×0.25 BkBlN horizontal (E); 0.25×0.5 NBN vertical (F) *versus* 0.5×0.25 NBN horizontal (G). In all these different treatments, proportions of the different parts were the same, e.g. treatments B and C, and F and G are made of 50% blue, 50% net, whereas black, blue and net are 25%, 50% and 25% respectively, in treatments D and E. This experiment was conducted only in Folonzo (Burkina Faso), for both *G. p. gambiensis* and *G. tachinoides*.

### Effect of Size

In this experiment, we wanted to know if smaller designs were more cost efficient than the standard. Hence three different sizes of targets, 0.75×0.5 (treatments C, L and M), 0.25×0.5 (F and H) and 0.25×0.25 (I and K) were investigated and compared to the standard. The experiment was undertaken on *G. p. gambiensis* in Orodara, Solenzo and Folonzo (Burkina Faso), on *G. tachinoides* in Folonzo, and on *G. p. palpalis* in Azaguié (Côte d'Ivoire), respectively with BkBlBk and NBN cloths. Only the treatment M was in BkBlN.

### The effect of replacing black colour by netting

The experiment was designed to determine if the black cloth could be replaced by the less expensive netting and, second, if it was necessary to place netting on both sides of the target or only one. This experiment also was performed on the 3 sites in Burkina and in Azaguié in Côte d'Ivoire.

### Measuring cost efficiency

The cost efficiency ratio of the devices was calculated and was expressed as the catch per unit area of one device divided by catch per unit area of the standard. Let us assume a target of 1 m^2^ catches 100 tsetse and a new design of 0.1 m^2^ also catches 100. So the tsetse/m^2^ for each target is 100 (100/1 = 100) and 1000 (100/0.1 = 1000). The improvement in cost efficiency would be viewed as being 1000/100 = 10 - ie we get 10x more tsetse per dollar/CFA/euro spent.

## Results

### Effect of shape

Horizontal-oblong targets caught consistently more tsetse than the vertical ones for both *G. p. gambiensis* and for *G. tachinoides* (see tables 1 and 2), for the three pairs which were compared (treatment C vs B, E vs D, G vs F). Results showed catch indices according to the ratio horizontal/vertical of 1.6, 3.5 and 4.5 respectively, with the two latter being significant at P<0.01 and P<0.001 respectively for *G. p. gambiensis* (Table 1). For *G. tachinoides,* these ratios were 1.60, 3.1 and 5 respectively (Table 2), these differences being highly significant for the two latter pairs (P<0.001) but not for the first pair in both sexes.

**Table 1:** Detransformed daily mean catches (transformed means in brackets) of *G. p. gambiensis* in Folonzo, Burkina Faso.

Target size (width×height) in meters	Material	Shape	Code	Rep	Male	Index	Female	Index	Total	Index
0.5×0.75	NBlN	V	B	14	3.0 (0.60)	0.57	3.3 (0.63)	0.46	6.5 (0.88)	0.50
0.75×0.5	NBlN	H	C	14	4.3 (0.72)	0.82	5.6 (0.82)	0.78	10.3 (1.05)	0.79
0.25×0.5	BkBlN	V	D	14	0.6 (0.21)	0.12	0.9 (0.27)	0.12	1.3 (0.37)***	0.10
0.5×0.25	BkBlN	H	E	14	2.8 (0.58)	0.54	1.7 (0.43)	0.24	4.5 (0.74)	0.35
0.25×0.5	NBlN	V	F	14	0.3 (0.10)***	0.05	0.5 (0.16)***	0.06	0.7 (0.24)	0.06
0.5×0.25	NBlN	H	G	14	2.2 (0.51)	0.42	1.4 (0.37)	0.19	3.5 (0.65)	0.27
			sed		0.085		0.084		0.090	
0.25×0.5	BkBlBk	V	H	24	0.9 (0.29)***	0.32	0.8 (0.25)***	0.25	1.8 (0.44)***	0.28
0.25×0.25	BkBlBk	V	I	24	0.3 (0.12)***	0.11	0.2 (0.08)***	0.07	0.5 (0.18)***	0.08
1×1	NBN	V	J	24	3.2 (0.62)	1.09	4.6 (0.75)	1.47	7.9 (0.95)	1.25
0.25×0.5	NBlN	V	F	24	0.7 (0.24)***	0.26	0.6 (0.19)***	0.18	1.4 (0.37)***	0.21
0.25×0.25	NBlN	V	K	24	0.1 (0.05)***	0.04	0.1 (0.05)***	0.04	0.2 (0.09)***	0.04
			sed		0.068		0.066		0.075	

Catches followed by *** differ from the standard at 0.001 level. Catch index is the mean catch of a target expressed as a proportion of that of the standard, which is 1*1 BkBlBk, see text for details.

Bk = Black; Bl = Blue; N = Net; H = Horizontal; V = Vertical.

Rep. = replicates.

**Table 2:** Detransformed daily mean catches (transformed means in brackets) of *G. tachinoides* in Folonzo, Burkina Faso.

Target size (width×height) in meters	Material	Shape	Code	Rep	Male	Index	Females	Index	Total	Index
0.5×0.75	NBlN	V	B	14	7.6 (0.094)	0.85	5.0 (0.78)	0.71	12.9 (1.14)	0.77
0.75×0.5	NBlN	H	C	14	11.9 (1.11)	1.32	8.1 (0.96)	1.14	20.7 (1.34)	1.23
0.25×0.5	BkBlN	V	D	14	0.8 (0.26)***	0.09	0.8 (0.25)***	0.11	1.6 (0.42)***	0.10
0.5×0.25	BkBlN	H	E	14	3.2 (0.32)***	0.35	1.9 (0.45)***	0.26	5.3 (0.80)***	0.31
0.25×0.5	NBlN	V	F	14	0.6 (0.21)***	0.07	0.3 (0.12)***	0.04	1.0 (0.29)***	0.06
0.5×0.25	NBlN	H	G	14	2.6 (0.56)***	0.29	2.0 (0.48)***	0.29	5.1 (0.78)***	0.30
			sed		0.086		0.086		0.086	
0.25×0.5	BkBlBk	V	H	24	0.7 (0.23)***	0.1	0.5 (0.17)***	0.09	1.1 (0.32)***	0.08
0.25×0.25	BkBlBk	V	I	24	0.5 (0.18)***	0.07	0.2 (0.09)***	0.04	0.8 (0.25)***	0.06
1×1	NBlN	V	J	24	8.2 (0.96)	1.15	9.2 (1.01)	1.73	17.9 (1.28)	1.39
0.25×0.5	NBlN	V	F	24	1.0 (0.29)***	0.14	0.9 (0.27)***	0.16	1.7 (0.43)***	0.13
0.25×0.25	NBlN	V	K	24	0.2 (0.08)***	0.03	0.2 (0.07)***	0.03	0.4 (0.14)***	0.03
			sed		0.062		0.062		0.062	

Catches followed by *** differ from the standard at 0.001 level. Catch index is the mean catch of a target expressed as a proportion of that of the standard, which is 1*1 BkBlBk, see text for details.

Bk = Black; Bl = Blue; N = Net; H = Horizontal; V = Vertical.

Rep. = replicates.

### Effect of size

For all three species in all the study areas, catches were highly correlated with size (P<0.001). As an example, in Solenzo while the catches of the standard were up to 18 flies/day for *G. p. gambiensis*, catches for targets H(0.25×0.5) and I (0.25×0.25) (both BkBlBk like the standard) were nearly 20 times less than the standard (p<0.001) (see Table 3). This ratio was almost the same with the netting treatments F(0.25×0.5) and K(0.25×0.25) where captures decreased to almost 0 tsetse/day, both for males and females *G. tachinoides* in Folonzo (see Table 2), and for *G. p. gambiensis* in Folonzo (Table 1) and Solenzo (Table 3). The same pattern was also observed in Côte d'Ivoire for *G. p. palpalis* (table 4) where fly densities were high.

**Table 3:** Detransformed daily mean catches (transformed means in brackets) of *G. p. gambiensis* in Solenzo, Burkina Faso.

Target size (width×height) in meters	Material	Shape	Code	Rep	Males	Index	Females	Index	Total	Index
0.25×0.5	BkBlBk	V	H	12	0.7 (0.24)	0.10	0.4 (0.14)	0.04	1.1 (0.31)	0.06
0.25×0.25	BkBlBk	V	I	12	0.4 (0.14)	0.05	0.4 (0.15)	0.04	0.7 (0.23)	0.04
1×1	NBlN	V	J	12	4.6 (0.74)	1.00	7.4 (0.92)	0.73	12 (0.11)	0.68
0.25×0.5			K	12	0.00 (0)	0.00	0.1 (0.3)	0.01	0.1 (0.03)	0.00
0.25×0.25			L	12	0.5 (0.19)	0.07	0.4 (0.13)	0.03	0.9 (0.27)	0.05
			sed		0.1		0.092		0.112	

Catches followed by *** differ from the standard at 0.001 level. Catch index is the mean catch of a target expressed as a proportion of that of the standard, which is 1*1 BkBlBk, see text for details.

Bk = Black; Bl = Blue; N = Net; H = Horizontal; V = Vertical.

Rep. = replicates.

**Table 4:** Detransformed daily mean catches (transformed means in brackets) of *G. p. palpalis* in Azaguié, Côte d'Ivoire.

Target size (width×height) in meters	Material	Shape	Code	Rep	Males	Index	Females	Index	Total	Index
0.25×0.5	BkBlBk	V	H	12	5.9 (0.84)***	0.3	4.3 (0.73)***	0.3	10.5 (1.06)***	0.3
0.25×0.25	BkBlBk	V	I	12	2.1 (0.49)***	0.1	0.5 (0.17)***	0.0	2.8 (0.58)***	0.1
1×1	NBlN	V	J	12	20.4 (1.33)	1.20	51.7 (1.78)***	3.60	77 (1.89)***	2.35
0.25×0.5	NBlN	V	K	12	4.2 (0.72)***	0.25	2.9 (0.59)***	0.20	6.9 (0.90)***	0.21
0.25×0.25	NBlN	V	L	12	0.8 (0.25)***	0.05	0.5 (0.18)***	0.03	1.5 (0.34)***	0.05
			sed		0.097		0.097		0.097	

Catches followed by *** differ from the standard at 0.001 level. Catch index is the mean catch of a target expressed as a proportion of that of the standard, which is 1*1 BkBlBk, see text for details.

Bk = Black; Bl = Blue; N = Net; H = Horizontal; V = Vertical.

Rep. = replicates.

One exception to this general trend was the 0.75×0.5 target which gave catch indices up to more than 0.8 (compared to 1 for the standard) for *G. p. gambiensis* (treatment C in table 1, treatments L, C, and M in Table 5) and up to 1.23 for the horizontal NBlN (treatment C) for *G. tachinoides* in Folonzo (table 2). For this particular size, there was no significant difference compared to the standard, whatever the colour combination.

**Table 5:** Detransformed daily mean catches (transformed means in bracket) of *G. p. gambiensis* in Orodara, Burkina Faso.

Target size (width×height) in meters	Material	Shape	Code	Rep	Males	Index	Females	Index	Total	Index
0.75×0.5	BkBlBk	H	L	12	9.6 (1.03)	0.99	8.4 (0.97)	0.73	18.9 (1.30)	0.86
0.75×0.5	NBlN	H	C	12	9.2 (1.01)	0.95	9.6 (1.03)	0.83	19.4 (1.31)	0.89
0.75×0.5	BkBlN	H	M	12	9.6 (1.03)	0.99	8.4 (0.97)	0.72	19.3 (1.30)	0.88
			sed		0.090		0.062		0.632	

Catch index is the mean catch of a target expressed as a proportion of that of the standard, which is 1*1 BkBlBk, see text for details.

Rep: Replicates.

Bk = Black; Bl = Blue; N = Net; H = Horizontal;

### Effect of replacing black colour with netting

For *G. p. gambiensis* in Folonzo and in Solenzo (tables 1 and 3), there was no significant difference in the catch of the 1×1 NBlN (J) compared to the standard. For the same species in Orodara (table 5), no difference appeared when comparing the three types of 0.75×0.5 targets (BkBlBk, NBlN, BkBlN) in terms of global catches (respective mean catches of about 20) and individually, none of them was significantly different from the standard (catch indices ≈0.9).

For *G. tachinoides*, the NBlN (0.75×0.5 (C) and 1×1 (J)) caught more than the standard with respective indices of 1.2 and 1.4, although these differences were not significant (table 2). For *G. p. palpalis* it appeared that the 1×1 NBlN (J) was significantly better than the standard (p<0.001) with an index of 2.4 (see Table 4).

### Landing behaviour

The general trend as regards landing behaviour is illustrated by experiment reported in table 5, where the following treatments were compared to the standard: (L) 0.75×0.5 BkBlBk, (C) 0.75×0.5 NBlN and (M) 0.75×0.5 BkBlN. All the targets had one blue coloured section and we compared the percentage of tsetse caught on that section. There was no significant difference in the landing responses of males and females and so the data were pooled. For all treatments, the blue section covered half the total area of the target but for only L was the catch (49%±3.0) close to the expected (50%). For all other treatments, the catches on the blue section were significantly less (20%±2.3, 30%±2.7, and 37±2.9 for A, C and M respectively). Thus the majority of tsetse were contacting either the the netting or black sections.

### Cost efficiency

The targets offering the best cost efficiency ratios for the three tsetse species studied are listed in table 6. For *G. p. gambiensis* the highest cost efficiency ratios were obtained with the horizontal 0.5×0.25 BkBlN (E) with a cost efficiency of 2.8, then with the 0.75×0.5 NBlN (C) and 0.75×0.5 BkBlN (P) both horizontal, with cost efficiency ratios respectively of 2.37 and 2.35. For *G. tachinoides*, the horizontal 0.75×0.5 NBlN (C) was the most cost-effective with a ratio of 3.28, followed by the 0.5×0.25 BkBlN (E) and NBlN (G) with respective ratios of 2.48 and 2.4. For *G. p. palpalis* the best was the vertical 0.5×0.25 BkBlBk (H) with a cost efficiency ratio of 2.4. Hence higher cost efficiency ratios (2–3) were achieved with targets smaller than the standard except for *G. p. palpalis*, and in all cases, including G. p. palpalis, it is worth noting the highest cost efficiency ratios were obtained with targets incorporating netting.

**Table 6:** Cost efficiency indices for the better designs.

Species	Target size (m× m)	Material	Shape	Code	Mean density (Both sexes)	Index	Tsetse/m2
	1×1	BkBlBk	V	A	-	1	1
*G.p. gambiensis*	0.5×0.25	BkBlN	H	E	4.5	0.35	2.8
*G.p. gambiensis*	0.5×0.25	BBlN	H	G	3.5	0.27	2.16
*G.p. gambiensis*	0.25×0.5	BkBlBk	V	H	1.8	0.28	2.24
*G.p. gambiensis*	0.75×0.5	BkBlBk	H	L	18.9	0.86	2.29
*G.p. gambiensis*	0.75×0.5	NBlN	H	C	19.4	0.89	2.37
*G.p. gambiensis*	0.75×0.5	BkBlN	H	M	19.3	0.88	2.35
*G. tachinoides*	0.75×0.5	NBlN	H	C	20.7	1.23	3.28
*G. tachinoides*	0.5×0.25	BkBlN	H	E	5.3	0.31	2.48
*G. tachinoides*	0.5×0.25	NBlN	H	G	5.1	0.30	2.4
*G.p. palpalis*	0.25×0.5	BkBlBk	V	H	10.5	0.3	2.4
*G.p.palpalis*	1×1	NBlN	V	J	77	2.35	2.35

Bk = Black; Bl = Blue; N = Net; H = Horizontal;

## Discussion

We demonstrate that catches increase with target size but that the increase is not in proportion to the increase in surface area. Hence, paradoxically, the numbers of tsetse killed per area of cloth, and by implication tsetse killed per dollar, decreases with increasing target size. This work has clearly shown, using blue/black coloured targets, that there is a correlation between target size and its attractiveness for several species of the Palpalis group. We demonstrate that improvements in cost efficiency of 2–3 fold can be achieved by using smaller targets than the one that is currently used routinely. With the targets investigated here, there is an optimum size between 50 and 75 cm, which if increased does not result in a significant increase in fly capture. In addition, it appeared that *G. p. gambiensis* and *G. tachinoides* were more attracted to horizontal than vertical devices.

While blue is mandatory for attractiveness, it does not elicit a strong landing response. Accordingly, the blue is accompanied by black cloth, which does induce landing [11], or netting which is invisible and cheaper, and hence flies collide with it inadvertently [23]. Hence a choice will have to be made regarding a trade off between cost and sustainability of using netting versus cloth in the manufacturing of targets. The consequences of these observations are crucial because they will allow the rational development of new cost-effective designs of target to control sleeping sickness and animal trypanosomiasis in Africa.

### Effect of Shape

Horizontal targets performed consistently better than vertical ones for both *G. p. gambiensis* and *G. tachinoides* in Burkina Faso. This confirms results previously reported for savannah flies of the Morsitans group in East and South Africa [17], [18]. However, this contrasts with observations on another species of the Palpalis group, *G. p. palpalis*, which has been reported to be more attracted to vertical rather than horizontal targets in forested area of Ivory Coast ([11], and I. Tirados *et al*., In Press.). Hence there appears to be consistent differences, even between species of the same group (i.e. Palpalis group here), regarding visual attraction to given shapes. The biological explanation is not precisely known so far, and may include several factors such as habitat, visibility, and/or feeding behaviour. It would be interesting to know if other tsetse species living in forest habitat would react like *G. p. palpalis* or not.

### Effect of size

We observed that catches were highly correlated with target size for the species studied, but only up to a maximum size of 75 cm wide. Hence when the minimum size threshold of ∼50 cm^2^ was reached, increasing the size of the target did not increase catches, as illustrated by the similar results of the horizontal 0.75×0.5 m target compared to the 1×1 m standard. These results are in general accordance with observations made for Morsitans group species in East and southern Africa, except that increasing size beyond 50 and 75 cm does not improve the catch. For *G. pallidipes*, attraction improved several fold as the width of visual panels increased from 25 to 200 cm, and the percentage of tsetse landing on visual panels before flying round increased up to several times with wider panels [19]. Recent studies in southern Africa also reported the correlation between size and catches for Morsitans group species [18].

Again here for size, as for shape above, knowing the exact cause of these differences between species is not obvious. Presumably it relates to the habitat or hosts of the flies. For instance it is generally acknowledged that savannah flies of the Morsitans group feed mainly on wild and domestic mammals, which are generally bigger than the reptiles which constitute the main diet of tsetse such as *G. fuscipes* [29]. *G. palpalis* and *G. tachinoides* are regarded as being opportunistic in their feeding habits, feeding on mammals, including humans, as well as reptiles. They may have developed a particular ability to detect small hosts.

### Effect of replacing black colour with netting, and landing behaviour

Although the three designs of horizontal target (0.75×0.5 BkBlBk, BkBlN and NBlN) were not significantly different regarding the total tsetse catches, replacing the black cloth by the black net consistently increased, albeit slightly, the catches of the different species. This confirms earlier observations of flies trying to avoid landing immediately on the blue cloth and then hit the “invisible” net when circling around the target [17].

The strongest landing response was elicited by blue and black targets, as reported previously [11], [14]. Although the difference was not always significant in our work in Orodara, males *G. p. gambiensis* were more attracted to the blue cloth while females landed more on the black. Differences in landing behaviour between males and females had been reported previously for studies of *G. p. palpalis* in Côte d'Ivoire [11], although in this study, as well as the one of Green [14], the reverse was observed, i.e. the proportion of males landing on black was greater than the one of females.

### A more cost effective new target design?

Regarding shape, the different trials showed that the horizontal target was always better when compared to the vertical for both *G. tachinoides* and *G. p. gambiensis.* When considering size, most flies were caught with the 0.75×0.5 cm targets, and also by the 0.5×0.25 cm. However especially for the latter, such small targets might be hidden by the dense vegetation found in humid savannahs and forested areas. More studies are underway to see if this is the case.

Using the flanking netting gave very promising results for the three species studied. Hence one could make a practical choice about the cost and durability of using netting vs. cloth in the construction of targets. It is noteworthy that Laveissiere *et al*. [11] also found that using net resulted in more catches than using black cloth. They however did not recommend its use for targets due to the local price of netting in Ivory Coast at that time. However, given that the price of black net is currently estimated to be 1/3 that of black cloth (T. Frandsen, pers. com.), changing the design of the BkBlBk 1 m^2^ target to a 0.75×0.5 NBlN target (see fig. 1) would increase cost efficiency by six fold (3 fold for cloth surface plus 3 fold for price of net vs black) without losing efficiency. It would have the further practical advantage of being effective for both *G. p. gambiensis* and *G. tachinoides* which are the main vectors of pathogenic trypanosomes to humans and domestic animals in West Africa, and which are found often together in savannah areas. For *G. p. palpalis* in Ivory Coast, although new insights have been brought by this study regarding their visual behaviour related to size of targets and the use of netting, it would be interesting to expand such studies to other countries where this tsetse species occurs.
